# Establishing the interpretability and utility of the 4-item BriefPCS

**DOI:** 10.1038/s41598-023-48433-6

**Published:** 2023-12-02

**Authors:** Dokyoung S. You, Karon F. Cook, Edward W. Lannon, Maisa S. Ziadni, Beth D. Darnall, Sean C. Mackey

**Affiliations:** 1 Department of Anesthesiology, Perioperative, and Pain Medicine, Stanford University School of Medicine, 1070 Arastradero Road, Suite 200, MC 5596, Palo Alto, CA 94304 USA; 2 Feral Scholars, 257 County Road 4754, Broaddus, TX 75929 USA

**Keywords:** Psychology, Health care

## Abstract

To reduce the patient burden associated with completing the 13-item Pain Catastrophizing Scale (PCS), the 4-item “BriefPCS” was developed. To date, no crosswalk has been developed that associates scores on the BriefPCS with PCS scores. Further, no study has compared the use of BriefPCS and PCS scores in a randomized clinical trial (RCT). We aimed to: (1) establish the interpretability of BriefPCS scores in reference to PCS scores, (2) compare the concurrent validity between the BriefPCS and PCS, and (3) asssess the use of BriefPCS in an RCT. First, we conducted equipercentile linking, created a crosswalk that associated scores of BriefPCS with PCS, and calculated differences between PCS and crosswalked PCS scores. Secondly, we compared Bootstrap correlation coefficients between PCS and self-reported measures of other domains. Lastly, we compared results from an RCT using BriefPCS scores versus PCS scores. Findings indicated that the correlation coefficient estimates with the BriefPCS and PCS scores were not significantly different. BriefPCS and PCS scores had similar ability to detect treatment-related changes. The BriefPCS scores validly, reliably, and accurately distinguish levels of pain catastrophizing. Additionally, the BriefPCS scores are sensitive to changes after behavioral interventions, with less respondent burden compared to the PCS scores.

## Introduction

Pain catastrophizing is a coping strategy characterized by a pattern of maladaptive cognitive and affective response to pain^1^. Evidence from pain research indicates that higher pain catastrophizing is associated with worse pain intensity ratings and greater disability, both concurrently^1–3^ and several months after self-reports of pain catastrophizing^4,5^. Reductions in pain catastrophizing are associated with lowered pain ratings, improved disability status, and increased likelihood of returning to work^6,7^. Psychological interventions have been developed that are effective in reducing pain catastrophizing^8,9^.

Currently, the most well-known measure of pain catastrophizing is the 13-item Pain Catastrophizing Scale (PCS). Substantial evidence has accumulated to support the validity of PCS scores, and the measure is widely used in research^1,10,11^. Completing the 13 items, however, can be burdensome to research participants, who often complete extensive survey batteries involving multiple measures, and to patients and staff at busy outpatient clinics. Patients seeking treatment are likely to be asked to report on several relevant health outcomes, in addition to pain catastrophizing, including pain intensity, depression, anxiety, pain interference, and physical and social function^12,13^. Short-forms, such as the 4-item BriefPCS, have been developed to reduce response burden and promote the utility of the PCS^14–16^. The four items of the BriefPCS were selected from the 13-item PCS based on triangulation of qualitative and quantitative evaluations of the items^14^.

One study compared the PCS and BriefPCS based on Rasch analysis and calculated test information for the PCS, the BriefPCS, and other short form scales^14–16^. Using Rasch analysis, the study identified four items with an acceptable fit, adequate unidimensionality, and adequate reliability. Test information estimates the degree to which a measure discriminates different levels of the outcome being measured^17,18^. However, test information is the sum of the information in all the items of the scale. Adding items to a measure will always increase the information sum and deleting them will decrease it. What is more relevant is how well the BriefPCS will function in a randomized controlled trial (RCT) compared to the PCS, and this has not been evaluated.

Based on these considerations, the current study aimed to characterize the validity, reliability, and accuracy of the BriefPCS in distinguishing levels of pain catastrophizing in adults with chronic pain who sought treatments at a tertiary pain clinic, and to characterize the BriefPCS’s sensitivity to change following pain psychology interventions. We hypothesized that PCS scores crosswalked from BriefPCS scores would accurately predict actual PCS scores. Furthermore, the concurrent validity of the BriefPCS would be similar to that of the PCS. Finally, we hypothesized that the BriefPCS would perform similarly to the PCS in a large RCT of pain psychology interventions in patients with chronic pain.

## Methods

**Figure 1:**
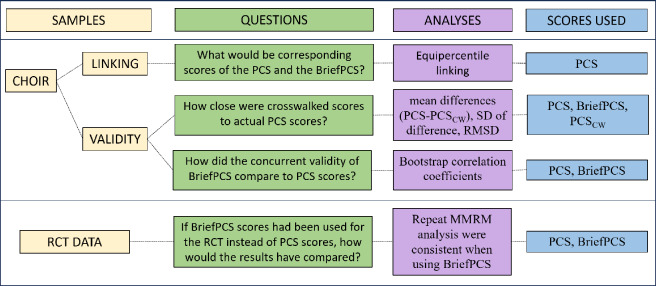
Research methodology diagram. *PCS* Pain Catastrophizing Scale, *PCS*_*CW*_ PCS scores crosswalked from BriefPCS, *RMSD* Root mean squared deviations, *MMRM* Mixed models for repeated measure.

Figure 1 summarizes the methodology used for this study and clarifies which samples and measures were used for the analyses of the study’s three research hypotheses.

### Samples

Study procedures, which involved exclusively retrospective review of clinical data, were approved by the Institutional Review Board at the Stanford University School of Medicine (IRB No. 28435). All methods were carried out in accordance with relevant guidelines and regulations. Informed consent for the standard care procedure and treatment at our clinic was obtained from all patients and their legal guardian. For the current study, we used data collected using CHOIR. CHOIR (http://choir.stanford.edu) is an open-source learning healthcare system that incorporates patient- and clinician reported outcomes across a variety of clinical domains, including pain intensity, physical and psychosocial function (including pain catastrophizing) and global health^19,20^. CHOIR administers both traditional long-form assessments (e.g., the PCS) and item response theory (IRT)-based assessments from the Patient-Reported Outcomes Measurement Information Systems (PROMIS) item banks developed by the National Institutes of Health. Data from CHOIR have been used in prior empirical work^21–27^; however, no publications have presented data extracted from CHOIR related to addressing the aims in this study.

We extracted self-report data from consecutive 21,226 adult patients with mixed etiology of chronic pain seeking treatment at a tertiary pain clinic collected by CHOIR. The data extracted were collected during the period of October 2014 to January 2021 and included responses to the PCS and measures from the PROMIS. Only the initial survey that participants completed was included for the current analysis. The extracted data were randomly divided into two halves to form a *Linking Sample* and a *Validation Sample*. Modeling equations, including linking equations, best fit the originating sample because they model all score variation, including random variation. Using an independent sample for linking and validation mitigates this effect, increasing the generalizability of the results. No missing values were found in PCS scores and a listwise deletion was used to examine the concurrent validity.

The third sample for this study was comprised of data collected for a previously published RCT in which PCS scores were the primary outcome, and other self-reported measures were secondary outcomes^8^. Hereafter, we refer to these data as the RCT Sample. In this RCT 263 adults with chronic low back pain were randomized to (1) single-session pain relief skills intervention (Empowered Relief; ER); (2) 8-session cognitive behavioral therapy (CBT) for chronic pain; or (3) single-session health and back pain education class (HE). At 3 months (the primary endpoint), empowered relief was found to be noninferior to CBT for pain catastrophizing scores as well as other self-reported outcomes.

## MEASURES

*The 13-item Pain Catastrophizing Scale* (PCS) was developed to measure the degree of pain catastrophizing in people with chronic pain^1^. Research has established the validity of the scores for this purpose. For example, in the developmental study, PCS scores had high internal consistency (Cronbach α = 0.87) and high test–retest reliability (*r* = 0.70 at 10-week interval)^1^. Each item is rated on a 5-point scale (0: not at all to 4: all the time) and the total scores range from 0 to 52, with higher scores indicating higher pain catastrophizing. The CHOIR measures time to complete each item of the 13-item PCS. We have found that it takes, on average, 110 s to complete the PCS.

*The BriefPCS* is a 4-item PCS short-form^14^ consisting of one item measuring helplessness (item 4 on the original PCS) and three rumination items (items 9, 10, 11). The BriefPCS is rated on a 5-point Likert scale, from 0 (not at all) to 4 (all the time), and the total scores range from 0 to 16. BriefPCS scores showed adequate fit to a unidimensional model and were highly correlated with PCS scores (*r* = 0.94)^14^. Bootstrapped Pearson’s *r* correlations between BriefPCS scores and scores on self-reported measures of other domains such as depression and pain interference supported their concurrent validity^14^. The magnitude of the score correlations when the BriefPCS was used were very similar to those calculated using PCS scores (magnitude differences $\le$ 0.04). Based on time measured for the 13-item PCS at an item level, estimated time to complete the 4-item BriefPCS is 36 s.

*Patient-Reported Outcomes Measurement (PROMIS)* The PROMIS-depression, anxiety^28^, average pain^29^, pain interference^30^, and physical function (upper extremity and mobility)^31^ measures are part of NIH’s PROMIS compendium of measures. These PROMIS measures were used to examine their relationship with the BriefPCS scores. All PROMIS measures were administered using computer adaptive testing to reduce response burden with sufficient precision^32,33^. The PROMIS uses T-score metric that is referenced to a sample matching the 2005 US Census population with respect to important demographic values (*M* = 50, *SD* = 10)^34^. Higher T scores indicate more of the symptom or outcome being measured.

### Research questions and analyses

In this study we addressed three different questions. To avoid confusion, we highlight the differences here. For the first question, we evaluated the accuracy of predicting PCS scores based on BriefPCS scores using a crosswalk. PCS scores were compared to PCS_CW_. The second question did not use crosswalked scores. We compared PCS and BriefPCS scores with respect to their responsivity and concurrent validity. Our last question was to examine the impact of using BriefPCS scores on RCT results. BriefPCS scores were used for the third question. We compared the RCT results between when using BriefPCS scores and PCS scores.

#### Question 1: can crosswalked BriefPCS scores accurately predict actual PCS scores?

##### Developing a crosswalk

Based on the Linking Sample, loglinear modeling was used to continuize and smooth the cumulative distributions of the observed scores of the BriefPCS and the PCS^35^. Percentile ranks of the scores were calculated. Scores with common percentile ranks were identified and used to estimate a curve that best described the relationship between scores of each measure^35^. The direction of linking was from the BriefPCS total scores to the PCS total scores. R package (“equate”) was used for this analysis^36^. A crosswalk table was created that associated scores of the BriefPCS with scores on the PCS.

##### Crosswalk accuracy

Within the Validity Sample, we used participants’ BriefPCS scores and the crosswalk to obtain crosswalk-predicted PCS scores, hereafter referred to as PCS_CW_ scores. We took actual PCS total scores as the criterion by which we evaluated the accuracy of the crosswalk, calculating the mean differences (PCS-PCS_CW_), standard deviation of difference, and root mean squared deviations (RMSD).

#### Question 2: how does the concurrent validity of BriefPCS scores compare to that of PCS scores?

To compare the concurrent validity of BriefPCS and PCS scores, we calculated Bootstrapped Pearson correlations between PCS scores and scores on PROMIS measures of pain intensity, pain interference, physical function (upper extremity movement, mobility), depression, and anxiety. We repeated these analyses using BriefPCS scores. Results were evaluated based on whether 95% CIs of coefficient estimates overlapped, which would indicate that coefficient estimate differences were not statistically significant (alpha ≤ 0.05)^14^.

#### Question 3: what would be the impact on RCT results of using BriefPCS scores instead of PCS scores?

The RCT Sample was used to repeat analyses conducted in the original published study, but using BriefPCS scores rather than PCS scores^8^. The details of the analyses used in the original study are published^8^. For our purposes, we targeted the primary outcome of pain catastrophizing at 3 months after treatment (the primary endpoint), operationalized in this study as BriefPCS scores rather than PCS scores. Mixed models for repeated measure (MMRM) regression analysis were conducted using baseline, 1-month, and 3-month scores. For all comparisons among ER, CBT, and HE in the original study, we recalculated the statistical tests using BriefPCS scores, and resulting p-values were compared to those reported for the original study. Of particular interest was whether any of the conclusions of the original study would have changed had BriefPCS been used instead of the PCS.

## Results

### Demographic and clinical characteristics

Table 1 summarizes the demographic and clinical characteristics for the Linking Sample (*N* = 10,613) and the Validation Sample (*N* = 10,613). Demographic and clinical characteristics were not significantly different between the two samples (*p* ≥ 0.165). Participants in both samples were predominantly female, white/Caucasian, married, and middle-aged. Both samples had a median education level of a bachelor’s degree. The mean average pain intensity rating was 5.4 for the two samples.

**Table 1:** Demographic and clinical characteristics of the linking and validation samples.

Demographic characteristics	Linking sample		Validation sample		*t*	*p*
	*N* = 10,613		*N* = 10,613			
		*M* (*SD*)		*M* (*SD*)		
Age		60.6 (16.9)		50.6 (16.8)	0.03	0.978

	*N*	(%)	*n*	(%)	*χ*^*2*^	*P*
Gender					0.36	0.835
Female	6916	(65.2)	6875	(64.8)		
Male	3338	(31.5)	3444	(31.8)		
Unknown/missing	359	(3.4)	360	(3.4)		
Race/ethnicity					2.89	0.823
White/non-Hispanic	5637	(53.1)	5698	(53.7)		
White/Hispanic	1099	(10.4)	1112	(10.5)		
Other	1018	(9.6)	951	(9.0)		
Asian	943	(8.9)	935	(8.8)		
Black/African American	377	(3.6)	368	(3.5)		
American Indian/Pacific Islander	109	(1.0)	112	(1.1)		
Missing/refused	1430	(13.5)	1437	(13.5)		
Marital status					1.52	0.958
Married	5834	(55.0)	5780	(54.5)		
Never married	2101	(19.8)	2149	(20.2)		
Divorced	1199	(11.3)	1176	(11.1)		
Living together	671	(6.3)	673	(6.3)		
Widowed	436	(4.1)	455	(4.3)		
Separated	228	(2.1)	234	(2.2)		
Missing	144	(1.4)	146	(1.4)		
Education					3.91	0.419
High school degree or less	1397	(13.2)	1369	(12.9)		
Some college or Associate degree	3395	(11.2)	3408	(32.1)		
Bachelor’s degree	2941	(27.7)	2860	(26.9)		
Master’s or higher degree	2727	(25.7)	2801	(26.4)		
Unknown/missing	153	(1.4)	175	(1.6)		

Clinical characteristics	*n*	*M* (*SD*)	*n*	*M* (*SD*)	*t*	*p*
Pain intensity (average)	10,612	5.4 (2.3)	10,612	5.4 (2.3)	0.23	0.820
PROMIS						
Depression T scores	10,305	53.0 (10.0)	10,332	52.9 (10.1)	0.72	0.471
Anxiety T scores	10,277	54.1 (9.9)	10,307	54.2 (10.0)	− 0.86	0.392
Pain interference T scores	10,463	63.0 (8.0)	10,472	63.0 (8.1)	1.39	0.165
Upper extremity T scores	7593	41.3 (10.7)	7457	41.4 (10.8)	− 0.32	0.725
Mobility	7570	42.5 (10.1)	7443	42.6 (10.0)	− 0.69	0.491
The PCS total scores	10,613	21.1 (12.7)	10,613	21.3 (12.9)	− 1.03	0.303
The BriefPCS total scores	10,613	7.1 (4.6)	10,613	7.1 (4.7)	− 0.79	0.431
The PCS_CW_ scores	10,613	21.1 (12.7)	10,613	21.2 (12.8)	− 0.74	0.459

*Race Other* multiracial or unknown, *PCS* Pain Catastrophizing Scale, *BriefPCS* the 4-item PCS, *PCS*_*CW*_ PCS scores crosswalked from BriefPCS scores.

### Can crosswalked Brief-PCS scores accurately predict actual PCS scores?

Table 2 is the crosswalk table for converting BriefPCS scores to their equivalent PCS scores based on equipercentile linking. The crosswalked PCS scored were highly correlated with actual PCS scores (*r* = 0.95). The mean difference (PCS-PCS_CW_) was 0.04, range = − 18.50 to 17.20. Both standard deviation of differences and RMSD values were 4.16. In the context of low back pain, minimal important change for the PCS has been estimated 8 points for those with scores < 30, and 11 points for score > 30^37^.

**Table 2:** Crosswalk tables for estimating PCS scores based on scores on the BriefPCS (linking sample *n* = 10,613).

Percentile	BriefPCS scores	PCS_CW_ scores	SE
6.6	0.0	1.6	0.14
12.5	1.0	4.9	0.18
18.7	2.0	7.7	0.19
26.2	3.0	10.3	0.21
35.5	4.0	12.8	0.22
42.3	5.0	15.4	0.23
48.9	6.0	17.9	0.24
55.4	7.0	20.6	0.25
62.8	8.0	23.2	0.26
68.7	9.0	25.9	0.28
74.3	10.0	28.5	0.30
79.1	11.0	31.2	0.30
85.0	12.0	33.8	0.32
88.7	13.0	36.5	0.34
92.1	14.0	39.5	0.36
95.0	15.0	43.1	0.35
100.0	16.0	48.4	0.31

PCS = Pain Catastrophizing Scale. Scores are rounded to the one decimal place. PCS_CW_ = PCS scores crosswalked from BriefPCS scores, SE: Standard Error of the PCS_cw_ Scores.

### How does the concurrent validity of BriefPCS scores compare to that of PCS scores?

Table 3 reports the 95% CIs of bootstrapped Pearson correlation coefficients, calculated in the validity sample, between PCS scores (i.e., PCS and BriefPCS) and scores on PROMIS measures (pain intensity, pain interference, physical function upper extremity and mobility, depression, and anxiety). There were no statistically significant differences in coefficient estimates with PROMIS scores based on the PCS and BriefPCS scores, as indicated by the overlaps of the 95% CIs.

**Table 3:** Intercorrelations among PCS scores, Brief PCS scores, and scores on PROMIS® measures (validation sample).

	*n*	PCS			BriefPCS		
		*﻿r*	95% CI		*r*	95% CI	
PCS		N/A			0.947	0.945	0.949
Average pain ratings	10,612	0.413	0.393	0.432	0.400	0.381	0.420
PROMIS							
Pain interference	10,472	0.554	0.537	0.570	0.538	0.521	0.555
Upper extremity	7457	− 0.325	− 0.346	− 0.303	− 0.304	− 0.325	− 0.283
Mobility	7443	− 0.308	− 0.328	− 0.286	− 0.295	− 0.317	− 0.273
Depression	10,332	0.599	0.582	0.615	0.559	0.542	0.575
Anxiety	10,307	0.587	0.570	0.603	0.556	0.538	0.572

Bootstrap with 5000 resampling, all *p* values < 0.001. *PCS* the 13-item Pain Catastrophizing Scale, *BriefPCS* the 4-item PCS.

### What would be the impact on RCT results of using BriefPCS scores instead of PCS scores?

Detailed demographic information for the RCT Sample has been published^8^. Briefly, the sample was 49.8% female, 60.2% White, 60.8% married; 27.4% of the sample held a bachelor’s degree or less. Like the Linking and Validity Samples, this longitudinal sample was predominately white and middle aged. The mean pain intensity rating was 4.6.

Table 4 presents comparisons for pain catastrophizing at months 1 to 3, adjusted for baseline pain catastrophizing scores and based on intention-to-treat analysis. The group comparison of p-values revealed that compared to HE, ER and CBT were significantly better in reducing pain catastrophizing at all timepoints after treatments and that ER and CBT were similar in reducing pain catastrophizing at 2 and 3 months after treatment. Therefore, the results and conclusions at Month 2 and 3 would be the same using the BriefPCS total scores instead of the PCS total scores^8^. However, the result at Month 1 (*p* value of 0.029) would be different from the original study (using α of 0.025 to determine the inferiority).

**Table 4:** Between-group differences in posttreatment pain catastrophizing as measured by the Pain Catastrophizing Scale (PCS) and the Brief Pain Catastrophizing Scale Scores (BriefPCS).

	Mean (SD) scores		Mean (SD) scores		Mean (SD) scores		Group comparison p-values					
	ER		CBT		HE		CBT vs HE		ER vs HE		CBT vs ER	
	PCS	BriefPCS	PCS	BriefPCS	PCS	BriefPCS	PCS	BriefPCS	PCS	BriefPCS	PCS	BriefPCS
	M (SD)	M (SD)	M (SD)	M (SD)	M (SD)	M (SD)	*p* value		*p* value		*p* value	
Baseline	22.09 (9.84)	7.46 (3.56)	23.01 (8.98)	7.46 (3.56)	24.81 (10.32)	8.17 (3.24)	N/A		N/A		N/A	
Month 1	15.49 (8.90)	5.57 (3.36)	12.53 (8.26)	4.31 (3.27)	19.98 (9.60)	6.81 (3.21)	< 0.001	< 0.001	0.005	0.020	0.009	0.029
Month 2	13.95 (9.78)	4.67 (3.54)	12.57 (8.09)	4.32 (2.78)	19.36 (10.32)	6.32 (3.52)	< 0.001	< 0.001	0.002	0.002	0.260	0.722
Month 3	13.17 (10.15)	4.69 (3.82)	11.87 (9.25)	3.87 (3.16)	19.74 (9.95)	6.53 (3.35)	< 0.001	< 0.001	< 0.001	< 0.001	0.340	0.232

*PCS* Pain Catastrophizing Scale, *BriefPCS* the 4-item PCS, *CBT* Cognitive Behavioral Therapy, *ER* Empowered Relief, *HE* Health Education.

## Discussion

Consistent with our study hypotheses, our findings indicate that the BriefPCS scores validly, reliably, and accurately distinguish levels of pain catastrophizing in adults with chronic pain seeking care at a tertiary pain clinic. In addition, the BriefPCS scores are sensitive to changes after pain psychology interventions, with substantially less respondent burden than the PCS. Our findings have important theoretical and clinical implications.

Our study's first aim was to develop a crosswalk table to associate BriefPCS scores with PCS scores. To our knowledge, this is the first study to use equipercentile linking to extend the interpretability of the BriefPCS scores. The crosswalk proved quite accurate in predicting mean PCS scores in the Validation Sample. The correlations between crosswalked PCS scores and actual PCS scores was high (0.95). The SD of the differences and the RMSD both had values 4.15. This is well below the clinically important difference values of 8 and 11 estimated for the PCS based on a low back pain sample^37^. A caveat, however, is the variability of the results at the individual level. Discrepancies in scores PCS-PCS_CW_ ranged from − 18.50 to 17.20. The use of cross-walked scores at the individual level (e.g., for clinical monitoring) or with smaller sample sizes may not be appropriate. For example, if a patient has been administered the PCS and the BriefPCS at different times over a treatment period, it would not be appropriate to evaluate the patient’s trajectory by comparing PCS and PCS_CW_ scores. A better approach would be to compute the individual’s BriefPCS score based on the PCS response. This would avoid the potential impact of linking error. Note, this caveat is not about the appropriateness of the BriefPCS scores in a clinical setting. Compared to PCS scores, the BriefPCS had comparable responsiveness and good construct validity as evidenced by their correlations with other self-report measures.

In our concurrent validity analysis, BriefPCS scores functioned quite well compared to PCS scores. Correlation coefficients for the association between BriefPCS scores and PROMIS scores were lower than those obtained using PCS scores, but not substantially. Some of this disparity may be due to the restriction of range in BriefPCS total scores compared to PCS total scores; in the former, there are 17 possible scores and, in the latter, there are 53. This difference may have attenuated the values of the correlation coefficients. BriefPCS and PCS scores were univocal on the relationship between pain catastrophizing and other relevant domains. Higher catastrophizing was moderately associated with greater pain intensity, pain interference, depression, and anxiety (*r*s = 0.400 ~ 0.599) and less physical function (*r*s = − 0.325 ~ − 0.295 for upper extremity movement and mobility). These results also concurred with those obtained in the study conducted to create and evaluate the BriefPCS^14^.

Of particular interest for clinical trials of pain intervention was our secondary analysis of a 3-arm RCT investigating the effectiveness of a single session ER and an 8-week CBT for pain in comparison to HE^8^. We found that our conclusions would have been unchanged if the study had used BriefPCS scores instead of PCS scores^8^. CBT and ER were significantly better at reducing pain catastrophizing than HE. The *p* values were smaller when the PCS scores were used, but incrementally so. There is a difference level between CBT, ER, and HE that, would have been statistically significant with PCS scores, but not with BriefPCs scores when setting *p* value at < 0.01. Based on the differences in p-values, however, the difference would have been quite small and the clinical relevance would be questionable. Nevertheless, the researcher is responsible for weighing the impact of response burden and increased precision for a particular research question and a specific setting. We concluded that the use of BriefPCS scores would be defensible for most such questions and contexts. Future research should compare the BriefPCS and PCS with regard to statistical power and impact on sample sizes to find different levels of treatment effect.

A few limitations should be noted in understanding our findings. First, we obtained our Linking and Validation Samples at a single tertiary pain clinic in Northern California, and thus, our results may not generalize to different clinical or community settings. Although our sample was inclusive of all patients seeking treatment at a tertiary pain clinic, our sample was mainly female, White/Caucasian, and highly educated people. Hence, the results from the present study may not generalize to male patients with chronic pain and individuals with ethic/racial diversity and low education levels. Yet, some evidence exist that the PCS is invariant across different clinical and non-clinical samples and across sex^38^. Secondly, the utility of BriefPCS will be limited for researchers interested in discriminating among the impact of the 3 sub-constructs of the PCS (i.e., magnification, helplessness, and rumination). Strength is a large sample size (*N* = 10,613 for linking and 10,613 for validation), which allowed us to investigate the full distribution of the BriefPCS scores in a large clinical sample. Our sample is approximately 25 times larger than the reference sample in the original study developing and validating the 13-item PCS (*N* = 851)^1^. Between these two samples, the means and standard deviations of the PCS total scores were similar (mean = 20.9 for the original sample and 21.1 for our sample; standard deviation = 12.5 for the original sample and 12.8 for our sample). Additionally, the respective 13-item PCS total scores of 10, 20, and 30 correspond to 25th, 50th, and 75th percentiles in the original^1^ and our samples. Therefore, our sample reproduces the distribution of the PCS total scores from the original sample to remarkable degree and our equipercentile linking results may have a high external validity.

Despite the limitations, our current study is the first to extend the interpretability of the BriefPCS scores using equipercentile linking to associate BriefPCS scores with scores on the PCS. The crosswalk could prove helpful in interpreting the results of research completed with the BriefPCS to the vast body of work that has been done using the PCS. Our results concluded that the BriefPCS is a viable alternative to the PCS, especially when clinicians and researchers have concerns about response burden. In conclusion, the BriefPCS is an efficient tool to assess pain catastrophizing and its responsiveness to pain psychology interventions.

## Data Availability

The datasets used and analyzed during the current study will be available from the corresponding author on reasonable request.
